# Pediatric Palliative Care Utilization Following Out-of-Hospital Cardiac Arrest at a Large US Quaternary Hospital

**DOI:** 10.3390/children13091142

**Published:** 2026-08-26

**Authors:** Suzanne R. Gouda, Emily J. Upham, Rachel D’Anna, Suzanne E. Dahlberg, Jennifer M. Snaman, Abby R. Rosenberg, Danielle D. DeCourcey

**Affiliations:** 1 Division of Medical Critical Care, Department of Pediatrics, Boston Children’s Hospital, 300 Longwood Avenue, Boston, MA 02115, USA; 2 Department of Supportive Oncology, Dana Farber Cancer Institute, Boston, MA 02115, USA; 3 Harvard Medical School, Boston, MA 02115, USA; 4 Biostatistics and Research Design Center, Boston Children’s Hospital, Boston, MA 02115, USA; 5 Division of Pediatric Hematology-Oncology, Department of Pediatrics, Boston Children’s Hospital, Boston, MA 02115, USA

**Keywords:** pediatric palliative care, out-of-hospital cardiac arrest, PICU, palliative care consultation

## Abstract

**Highlights:**

**What are the main findings?**

• In this 13-year retrospective cohort study of 126 patients, subspecialty pediatric palliative care (SPPC) was consulted in 18.3% of cases and occurred more often for Black patients and those with more pre-existing complex chronic conditions.
• Consultations occurred a median of 8 days after admission and addressed goals of care, medical decision making, and psychosocial support.

**What are the implications of the main findings?**

• SPPC consultation remains at low rates and occurs later in the illness course.
• Pediatric out-of-hospital cardiac arrest (OHCA) patients may benefit from standardized, proactive referral criteria to ensure equitable and comprehensive support for families.

**Abstract:**

**Background/Objective:** Pediatric out-of-hospital cardiac arrest (OHCA) carries high morbidity and mortality, placing immense psychosocial and sudden decision-making burdens on families. Subspecialty pediatric palliative care (SPPC) can provide crucial support for these multifaceted needs, but its utilization and timing in this population remain poorly characterized. This study aimed to characterize the current rate, timing, and care domains addressed by SPPC utilization in pediatric patients admitted to the pediatric intensive care unit (PICU) following OHCA. **Methods:** This was a 13-year retrospective cohort study (2012–2024) conducted at Boston Children’s Hospital, a quaternary academic children’s hospital. This study included 126 patients up to 21 years of age who experienced an OHCA and were admitted to a PICU for at least 24 h. Primary outcomes included the rate of SPPC consultation, timing of the initial consultation, and documented domains of care addressed. Exploratory correlates included patient demographics and clinical factors associated with consultation. **Results:** Among 126 patients included, 23 (18.3%) received an SPPC consultation. SPPC utilization was significantly higher for Black or African American patients (47% of the SPPC group vs. 9.9% of the non-consult group) and patients with a greater number of pre-existing complex chronic conditions (median: 1 vs. 0). The SPPC cohort had a longer observed median PICU length of stay (22 days vs. 5 days) and hospital length of stay (36 days vs. 6 days) in unadjusted comparisons. The median time from admission to the first SPPC consult was 8 days. When consulted, SPPC addressed goals of care in 96% of cases, medical decision making in 83%, and psychosocial support in 74%. **Conclusions:** SPPC is not often utilized and is typically introduced later in the illness course during the care of children following OHCA, often associated with patients with prolonged or complex clinical courses. The significant demographic-associated variances in consultation rates highlight the risks of subjective referral practices, underscoring the need for standardized, proactive integration of palliative care to support all families navigating this devastating event.

## 1. Introduction

Pediatric out-of-hospital cardiac arrest (OHCA) confers high morbidity and mortality. Every year, more than 7000 children experience OHCA across the United States [1,2]. Survival rates are poor following pediatric OHCA, ranging from 6% to 38% [3,4,5]. Less than 10% of patients will survive to discharge from the pediatric intensive care unit (PICU) with a favorable neurologic outcome [3,6]. Given the short- and long-term neurological, cognitive, emotional, and social morbidities, OHCA has become one of the top 10 causes of pediatric annual disability-adjusted life years lost in the United States [7]. Families of children who experience OHCA face sudden, critical decisions regarding life-sustaining therapies, while also grieving the loss of the life they expected for their child. For survivors, ongoing “integrated medical, rehabilitative, caregiver, and community support across the months to years after their cardiac arrest” is recommended [1]. Parents of non-survivors following OHCA experience high rates of complicated grief, with sudden or unexpected child death being an independent risk factor for grief-related psychiatric disorders [8]. Furthermore, families experience substantial and long-lasting emotional and psychological distress after hospital discharge, regardless of survival or significant changes in functional outcomes [9,10].

Subspecialty pediatric palliative care (SPPC) can address the multi-faceted needs of children and their families following an OHCA by providing additional support, including pain/symptom management, delineation of goals of care, complex medical decision making, psychosocial assessment, and bereavement care [11,12,13]. Early SPPC integration improves child and family outcomes in other serious illness settings and is recommended by the American Academy of Pediatrics [14,15,16,17]. However, despite clear benefits and guideline recommendations, SPPC remains historically underutilized in the post-arrest PICU population. A recent study using the PHIS database suggested only 13% of children who experienced OHCA received SPPC consultation [18]. Furthermore, when SPPC is involved, little is known about when or how it serves families and medical teams, which cannot be elucidated from large dataset studies given the granularity of data required. Our previous work described clinical and demographic characteristics associated with SPPC consultation at a US PICU and nationally, but was unable to describe referral indications, content of consultation, and frequency of SPPC involvement [18,19]. To address this gap and build upon this work, we conducted a single-site, retrospective cohort study using granular chart-level characterization to describe the current rate, timing, frequency, and care domains addressed for SPPC utilization in pediatric patients admitted to the PICU following OHCA.

## 2. Methods

### 2.1. Study Design and Setting

We conducted a 13-year retrospective cohort study (2012–2024) of pediatric patients admitted to Boston Children’s Hospital (BCH) PICU following an out-of-hospital cardiac arrest. BCH is a quaternary academic children’s hospital with multiple pediatric intensive care units (a cardiac ICU, a medical ICU, and a medical–surgical ICU) that provide post-arrest care.

During the observation period, BCH’s SPPC team was staffed by two to three full-time nurse practitioners, two to three full-time social workers, and up to 11 part- and full-time physicians. Any child with a potentially life-limiting medical condition was eligible for SPPC consultation, with no structured referral criteria. Consults were requested at the sole discretion of the primary medical team and/or family through the hospital’s electronic health record and/or paging system, staffed 24/7. The team followed patients both inpatient and outside the hospital, including ambulatory and virtual visits.

### 2.2. Participants

We included patients up to 21 years old who experienced an OHCA (defined as pulselessness requiring chest compressions for >one minute) and were admitted to any BCH PICU with a hospital length of stay (LOS) greater than 24 h between 1 January 2012 and 31 December 2024. For patients with multiple OHCA admissions (n = 2), secondary admissions were excluded to ensure all observations were independent of one another. The 24-h minimum hospital LOS inclusion criterion was chosen to allow for declaration of clinical trajectory to take place and a reasonable time interval appropriate for consultation of SPPC services. The age cutoff was selected due to BCH being the referring regional cardiac arrest center for patients up to 21 years of age.

### 2.3. Measurements

We queried the institutional electronic medical record for all patients with any primary and/or admitting diagnosis code relating to cardiac arrest during the defined study period. All available pre-admission documentation, inpatient medical records from index admission, and post-discharge encounters (ambulatory visits and/or hospital readmissions) up to one year were manually reviewed by two trained clinical research investigators (S.G. and E.U.) to extract patient demographics, pre-admission medical history, receipt and content of PPC consults, disease severity and medical intensity measures, invasive procedures, new medical technology, discharge disposition, code status, and mode of death (when applicable), with all data documentation completed in encrypted fire-wall protected case report forms. The first 25 charts were reviewed independently (S.G. and E.U.) and compared for inter-rater consistency, with greater than 95% agreement achieved. The remainder of eligible charts’ data elements were extracted by one study member (E.U.) and reviewed by the full study team (S.G. and E.U.).

Patient pre-existing comorbidities were identified and categorized according to Feudtner et al.’s complex chronic condition (CCC) classification system using codes from the International Classification of Diseases, 10th Revision, Clinical Modification [20]. Mode of death was classified into four categories determined by end-of-life care documentation, including (1) discontinuation of life-sustaining therapies; (2) non-escalation of care, defined as withholding new therapies or interventions while continuing current ones; (3) unsuccessful cardiopulmonary resuscitation; and (4) death by neurologic criteria.

For our primary outcomes regarding SPPC utilization, we collected metrics including prior SPPC involvement, the presence of an inpatient SPPC consult, frequency of SPPC family-facing visits, the timing of the initial and last consultation (measured in days from admission to first consult, and days from final consult to discharge or death), and the domains of care addressed during initial consultation per note author documentation (categorized as goals of care, medical decision making, and/or psychosocial support). We divided the cohort into two groups based on the presence or absence of SPPC consultation during admission for comparative analysis.

### 2.4. Statistical Analysis

Descriptive statistics were used to summarize patient demographic and clinical characteristics, overall and by SPPC utilization. All categorical variables were compared using Fisher’s exact tests, and continuous variables were compared using Wilcoxon rank-sum tests. All tests were two-sided with a type I error rate of 0.05, and no adjustments were made for multiple comparisons. Categorical variables were reported as frequencies and proportions; continuous variables were reported as medians (Q1 and Q3). No imputation of missing data was performed. A multivariable Firth’s penalized logistic regression model for SPPC consultation was conducted with a limited number of statistically significant and clinically meaningful characteristics that occurred prior to initial SPPC consultation to prevent model overfitting. All analyses were completed using R version 4.5.3.

## 3. Results

### 3.1. Overall Cohort Characteristics

**Figure 1:**
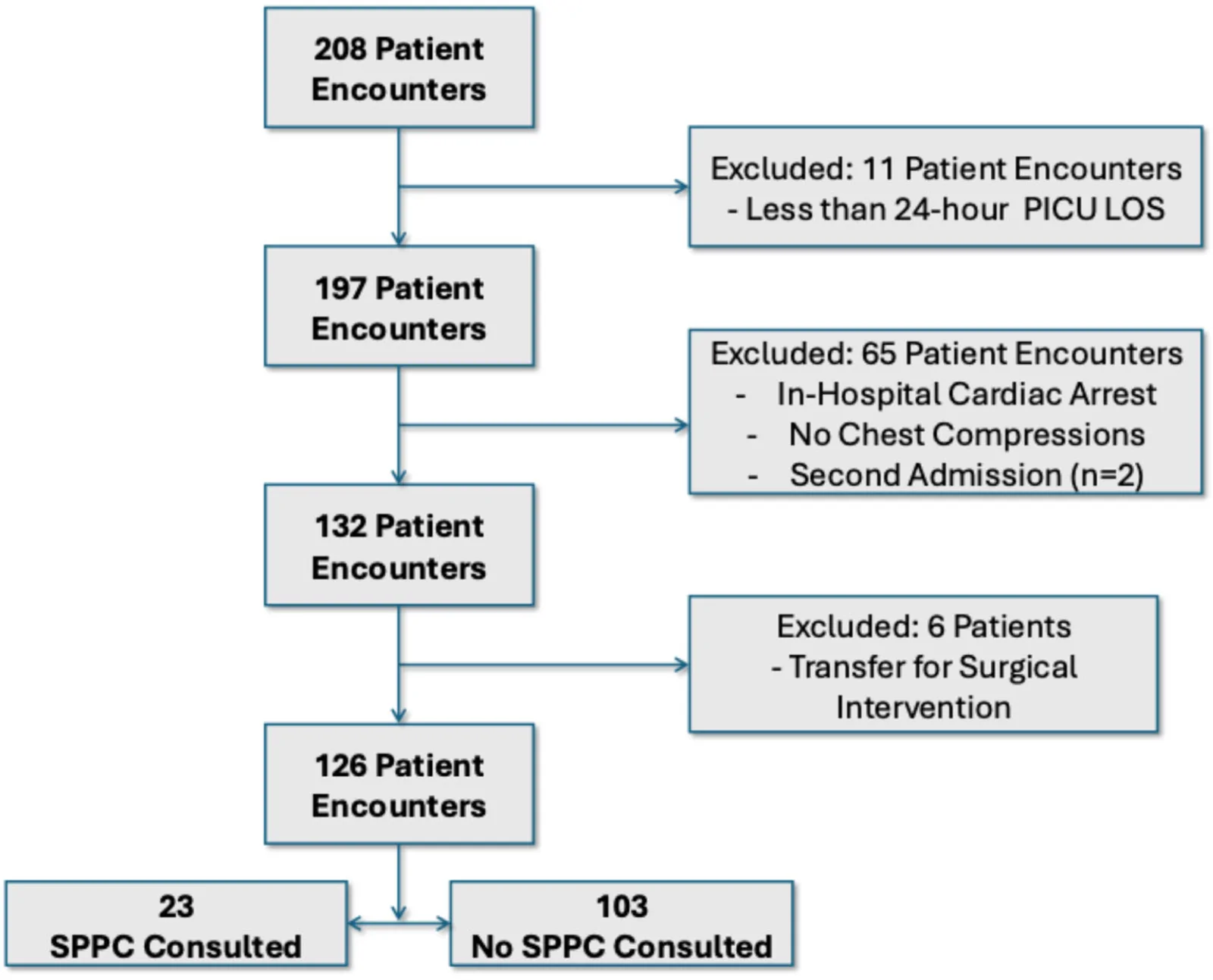
Chart review flowchart.

A total of 208 patient encounters were reviewed, and 126 pediatric OHCA patients met the inclusion criteria. A total of 63 patient encounters were excluded for lack of clear documentation of chest compressions received or in-hospital cardiac arrest. An additional two patient encounters were excluded for being a patient’s second OHCA admission. A further 11 patient encounters were excluded for hospital LOSs of less than or equal to 24 h. These 11 patients were 55% (6/11) male and mostly white (6/9), with a pre-existing CCC rate of 91% (10/11) and a mortality rate of 100% (11/11). Four of these patients excluded for stays of less than 24 h had prior involvement of SPPC (36%), and two of these patients had a consult during their admission (18%). An additional six patient encounters were excluded as they were transferred to BCH for higher-level surgical interventions only. For our primary outcome, SPPC was consulted in 23 of the 126 cases, representing a utilization rate of 18.3% (Figure 1). The median age in the overall cohort was 4.6 (IQR: 0.9, 14.0) years. Sixty-seven percent (85/126) were male, 61% (61/100) white, and 82% (87/106) non-Hispanic; 89% (112/126) preferred English as their primary language; and 64% (69/107) endorsed a religious preference of Christianity. Forty-one percent (52/126) of patients had at least one documented CCC, and 18% (23/126) were supported by medical technology prior to OHCA.

### 3.2. Demographic and Clinical Factors Associated with SPPC Consultation

There were significant differences in SPPC utilization based on patient race and number of prior CCC diagnoses. Black or African American patients received SPPC consults at disproportionately higher rates, with a rate of 52.9% (95% CI: 27.8–77%), while white patients had a much lower rate of 9.8% (95% CI: 3.7–20.2%). Of note, 26 patients did not have race documented. Additionally, the SPPC group had a greater number of pre-admission CCCs, with a median of one vs. zero in the non-SPPC group (*p* = 0.023). We found no significant differences in SPPC utilization based on age, gender, ethnicity, primary language, religious preference, or insurance status (Table 1).

Patients with certain specific categories of pre-existing CCCs were more likely to receive an SPPC consultation compared with children without those baseline conditions. These CCC categories significantly associated with SPPC consultation following OHCA included prior cardiovascular (35% [8/23] vs. 14% [14/103]; *p* = 0.029), gastrointestinal (35% [8/23] vs. 13% [13/103]; *p* = 0.025), congenital/genetic (30% [7/23] vs. 8% [8/103]; *p* = 0.007), and respiratory (26% [6/23] vs. 9% [6/103]; *p* = 0.031) complex chronic conditions. Neurologic/neuromuscular, renal, neonatal, hematologic/immunologic, malignancy, and metabolic complex chronic conditions did not demonstrate statistically significant differences between groups (Table 2).

Several clinical severity markers, interventions, and outcomes were significantly associated with receiving an SPPC consultation. The SPPC cohort demonstrated a markedly prolonged median PICU LOS (22 days vs. 5 days; *p* < 0.001) and overall hospital LOS (36 days vs. 6 days; *p* < 0.001). Patients in the SPPC cohort experienced significantly longer median days supported by mechanical ventilation compared with those who did not receive a consultation (19 days vs. 4 days; *p* < 0.001). ECMO utilization was significantly higher in the SPPC group (22% [5/23] vs. 5.8% [6/103]; *p* = 0.029). Furthermore, patients receiving an SPPC consult were significantly more likely to undergo placement of a new surgical feeding tube (39% [9/23] vs. 11% [11/103]; *p* = 0.002) and/or tracheostomy (22% [5/23] vs. 2.9% [3/103]; *p* = 0.005) during their admission. In-hospital mortality did not significantly differ between the two groups, with a mortality rate of 39% (9/23) in the SPPC group compared with 50% (51/103) in the non-SPPC group. Discharge disposition and mode of death (for non-survivors) were examined, neither of which reached statistical significance (Table 3).

A multivariable Firth’s logistic regression model was conducted using relevant pre- initial SPPC consult variables. Patients of Black/African American race have higher odds of receiving a consult when compared with white participants (OR: 20.3; 95% CI: 4.3–97.1), after adjusting for other variables, although the wide confidence interval suggests a level of uncertainty in the magnitude of the association. Patients categorized as other race also had higher odds of having a consult than white participants (OR: 4.8; 95% CI: 1.0–22.5). For each additional CCC, patients have approximately double the odds of receiving a consult (OR: 1.8; 95% CI: 1.1–3.0). Patients with previous SPPC involvement had higher odds of receiving a consult (OR: 4.2; 95% CI: 0.5–35.7), although this association was not statistically significant after adjustment (Table 4).

### 3.3. SPPC Utilization, Timing, and Scope

During the index hospitalization following OHCA, SPPC was consulted in 23 of the 126 cases, representing an average utilization rate of 18.3%. Of those cases, 74% of patients (17/23) received SPPC consultation for the first time in their medical care. Among the 23 patients who received SPPC, the median time from PICU admission to the first SPPC consult was 8 days (IQR: 2, 13). Of the patients with SPPC involvement who survived (n = 14), the median time from the first SPPC consult to hospital discharge was 36 (IQR: 13–61) days. For the subset of patients who died during the admission (n = 9), SPPC was consulted near the end of life, with a median time from first consult to death of only 3 (IQR: 2–9) days. Of note, three patients had prior SPPC involvement but did not receive SPPC services on the index admission. In those cases, the SPPC team had concluded their services in discussion with the families who no longer had active palliative care needs prior to each OHCA admission and did not get reconsulted (Table 1). The documented domains of care addressed during initial SPPC consultation (n = 23) included elicitation of goals of care discussions in 96% (22/23) of cases, medical decision-making support in 83% (19/23) of cases, and psychosocial support for the family in 74% (17/23) of cases, often with all three addressed (Table 5). All patients who received SPPC consultation had documentation of their goals of care. Most families identified seeking quality of life as their primary goal, prioritizing their child’s comfort (8/23, 35%), or prioritized balancing both life extension and quality of life together (12/23, 52%). For the patients who had SPPC involvement throughout admission, the team averaged 0.28 visits per hospital day, or one SPPC visit for every 3.6 hospital days. Additionally, 14 of the 66 (21%) patients from the total cohort who survived the OHCA admission received SPPC consultation within one year after discharge from the index PICU admission.

## 4. Discussion

This 13-year, single-site, retrospective cohort study highlights a profound gap in the delivery of comprehensive, family-centered care following pediatric OHCA. We found an inpatient SPPC consultation rate of only 18.3%, demonstrating that SPPC has low utilization for a patient population facing extraordinary morbidity, mortality, and long-lasting psychosocial distress [18]. In the minority of patients who received SPPC services, we found significant demographic variances, specifically with black families and those with pre-existing medical complexity. Additionally, initial SPPC consultations occurred a median of 8 days into the hospital course, potentially reflective of a responsive rather than proactive consultation culture. When engaged, SPPC provided intensive, multidimensional, longitudinal support, addressing multiple care needs simultaneously, including goals-of-care discussions, medical decision making, and psychosocial support for families. These novel findings show families have significant SPPC needs that are ongoing throughout and extending well beyond the index hospital stay, with a majority navigating post-arrest care without this added support. These findings extend prior work using large administrative datasets, which have demonstrated low rates of SPPC utilization following pediatric OHCA but have been unable to characterize how and when SPPC is integrated into care.

The demographic variances in SPPC allocation identified in this study demand careful exploration. Our finding, though exploratory, of black/African-American-identifying families receiving a disproportionately higher rate of SPPC consultation is notable and inconsistent with prior studies of palliative care utilization in both pediatric and adult populations [21,22]. Several explanations are possible, including differences in cohort composition, illness severity, or referral patterns within this single institution. However, these findings raise important questions about reliance on clinician-driven referral processes and the potential for how SPPC needs are determined. Some possible explanations include implicit biases that may influence which families clinicians perceive as “needing” extra support in communication and medical decision making, or conversely, which families clinicians feel most comfortable providing primary palliative care [21,22,23]. Standardized patient-facing tools and objective referral criteria have been shown to improve palliative care access and may help reduce unwarranted variation, promoting more consistent access across patient populations [24]. Due to the limitations of our retrospective data, we cannot determine whether SPPC referrals were clinically indicated, equitable, beneficial, and/or supportive for families. Regardless, addressing potential disparities in SPPC referral practices is essential to fostering trust and ensuring that SPPC is perceived as a supportive, rather than a mechanism to influence care, and warrants future exploration.

The timing of SPPC consultation in this cohort highlights a missed opportunity for earlier support. Families of children with OHCA must navigate sudden, high-stakes decisions in the setting of profound prognostic uncertainty, often within the first days of admission. In this study, most families did so without SPPC support during this critical period. Instead, SPPC was more commonly introduced later in the course, often associated with prolonged critical illness, ECMO utilization, evolving chronic complexity with newly acquired life-sustaining medical technologies (i.e., tracheostomies and surgical feeding tubes), or end-of-life care. Although we cannot establish causality, this could imply that referring PICU clinicians may be utilizing SPPC services for the subset of patients who have survived the initial acute post-arrest insult but are transitioning into a state of potentially new and/or significantly increased chronic medical complexity. While SPPC may play an important role for this cohort, allocating SPPC consultation for patients with chronicity could represent a missed opportunity. Early, proactive SPPC integration could provide crucial early grief support, longitudinal relationship building, and communication support for *all* families, regardless of whether the child’s ultimate trajectory is long-term technology dependence or end-of-life care and continued bereavement support. Furthermore, we found that pre-existing CCCs were a major driver of SPPC consultation. This likely reflects clinician recognition that these patients have established medical complexity and may benefit from additional support. For these families, an OHCA often forces a sudden, urgent reassessment of baseline goals of care, which appropriately prompts intensivists to engage SPPC. However, previously healthy children who experience a sudden OHCA face an equally devastating, albeit different, shift in their health trajectory, with equally profound needs. For these families, the abrupt transition from health to critical illness may be particularly destabilizing, underscoring the importance of equitable access to communication and psychosocial support regardless of baseline health status.

As our study demonstrated, when involved, SPPC addressed multiple care domains and the care delivered was comprehensive and longitudinal. SPPC teams consistently engaged in goals-of-care discussions and provided clear documentation of those goals, supported complex medical decision making, and addressed psychosocial needs. Notably, the majority of families articulated goals that balanced life-prolonging therapies with quality-of-life considerations, reflecting nuanced and individualized priorities rather than a singular focus on either life extension or comfort. This key finding directly upends the common misconception that SPPC involvement is only for end-of-life care or when goals are focused on comfort alone [25]. In addition, SPPC teams remained actively involved throughout hospitalization, with repeated visits over time, suggesting that their role extends beyond a single consultation to ongoing partnership with families and clinical teams.

This study has several limitations. It is a single-center, retrospective chart review conducted at a quaternary academic institution; therefore, the SPPC utilization rates and consultation practices may not be broadly generalizable to community or smaller academic PICUs. It is also important to note that because no adjustments were made for multiple comparisons, the results should be interpreted carefully due to the potential for inflated type I error. Additionally, retrospective EHR abstraction cannot capture the rationale behind clinician decisions to consult or not consult SPPC, nor can it assess the quality or depth of the primary palliative care provided by the PICU team. Detailed arrest characteristics such as downtime, initial rhythm, etc., also cannot be reliably abstracted from the EHR due to inconsistent and unreliable documentation practices, resulting in residual confounding. In those with SPPC consultation, chart review cannot capture the quality of communication, the family experience, or the degree to which SPPC impacted clinical care and trajectory. Furthermore, SPPC teams often provide support to primary medical teams in addition to the patients and families. Providers caring for pediatric OHCA can experience significant moral and emotional distress, which SPPC teams often tend to in the background of their family-facing work. This care role is rarely documented and difficult to abstract and/or quantify from medical records and, therefore, cannot be commented on in this study. Further qualitative work is needed to examine clinician and family perspectives and experiences of post-arrest care and the benefits and/or burdens of SPPC involvement in depth. Finally, the exclusion of patient encounters with a hospital LOS of less than 24 h, chosen to account for the feasibility of consulting SPPC in such a short time window, may introduce survivor selection and opportunity-for-consultation bias. Consequently, mortality and missed acute palliative care needs may be underrepresented.

## 5. Conclusions

SPPC remains underutilized and is introduced later in the illness course during the care of children and families navigating the devastating aftermath of pediatric OHCA. Current SPPC referral practices are not standardized, with consultation often allocated for patients with prolonged or complex clinical courses. Significant demographic variances in consultation rates, although exploratory, further underscore the potential risks of relying on non-standardized referral mechanisms. Earlier and more consistent integration of SPPC may better support families during periods of acute uncertainty and decision-making. Future work should focus on developing and testing strategies for earlier, consistent, and family-centered integration of palliative care, including objective, family-informed referral criteria and care delivery pathways. The impact of equitable and comprehensive palliative care support for all families facing the devastating and long-lasting impact of pediatric OHCA should be explored.

## Figures and Tables

**Table 1:** Patient demographics by SPPC consult status.

Characteristic	Overall ^a,b^ N = 126	SPPC Consult ^a,b^ N = 23	No SPPC Consult ^a,b^ N = 103	*p*-Value ^b,c^
Age at admission (years)	4.6 (0.9, 14.0)	3.9 (0.9, 12.0)	5.0 (0.9, 14.5)	0.6
Gender				0.092
Male	85 (67%)	12 (52%)	73 (71%)	
Female	41 (33%)	11 (48%)	30 (29%)	
Race				<0.001
White	61 (61%)	6 (32%)	55 (68%)	
Black or African American	17 (17%)	9 (47%)	8 (9.9%)	
Asian	8 (8.0%)	2 (11%)	6 (7.4%)	
American Indian or Alaska Native	1 (1.0%)	0 (0%)	1 (1.2%)	
Multiple Races	1 (1.0%)	1 (5.3%)	0 (0%)	
Other	12 (12%)	1 (5.3%)	11 (14%)	
Missing/prefer not to answer	26	4	22	
Ethnicity				0.4
Hispanic or Latino	19 (18%)	2 (9.5%)	17 (20%)	
Not Hispanic or Latino	87 (82%)	19 (90%)	68 (80%)	
Missing/prefer not to answer	20	2	18	
Primary language				0.3
English	112 (89%)	20 (87%)	92 (89%)	
Spanish	4 (3.2%)	0 (0%)	4 (3.9%)	
Arabic	3 (2.4%)	0 (0%)	3 (2.9%)	
Portuguese	2 (1.6%)	1 (4.3%)	1 (1.0%)	
Other	5 (4.0%)	2 (8.7%)	3 (2.9%)	
Religious preference				0.3
Christian denomination	69 (64%)	17 (77%)	52 (61%)	
Jewish	3 (2.8%)	1 (4.5%)	2 (2.4%)	
Muslim	5 (4.7%)	0 (0%)	5 (5.9%)	
Other	30 (28%)	4 (18%)	26 (31%)	
Missing/prefer not to answer	19	1	18	
Insurance status				0.5
Public	45 (37%)	8 (35%)	37 (37%)	
Private	60 (49%)	10 (43%)	50 (51%)	
Combination	17 (14%)	5 (22%)	12 (12%)	
Missing/prefer not to answer	4	0	4	
Pre-existing complex chronic conditions				0.11
Yes	52 (41%)	13 (57%)	39 (38%)	
No	74 (59%)	10 (43%)	64 (62%)	
Number of complex chronic conditions	0 (0, 2)	1 (0, 4)	0 (0, 1)	0.023
any previous technology dependence				0.13
Yes	23 (18%)	7 (30%)	16 (16%)	
No	103 (82%)	16 (70%)	87 (84%)	
Types of previous technology dependence				
Tracheostomy	12 (9.5%)	4 (17%)	8 (7.8%)	0.2
Surgical feeding tube	20 (16%)	8 (35%)	12 (12%)	0.011
NIPPV	3 (2.4%)	1 (4.3%)	2 (1.9%)	0.5
Home vent	11 (8.7%)	4 (17%)	7 (6.8%)	0.11
Cardiac device	3 (2.4%)	0 (0%)	3 (2.9%)	>0.9
Neurosurgical device	1 (0.8%)	0 (0%)	1 (1.0%)	>0.9
Surgical central line	1 (0.8%)	0 (0%)	1 (1.0%)	>0.9
Dialysis	0 (0%)	0 (0%)	0 (0%)	>0.9
Previous SPPC involvement				0.001
Yes	9 (7.1%)	6 (26%)	3 (2.9%)	
No	117 (93%)	17 (74%)	100 (97%)	

^a^ Median (Q1, Q3); n (%). ^b^ Missing values were excluded from percentage calculations and statistical tests; ^c^ Wilcoxon rank-sum test; Fisher’s exact test.

**Table 2:** Pre-existing complex chronic conditions by SPPC consult status.

	SPPC Consult N = 23 ^1^	No SPPC Consult N = 103 ^1^	*p*-Value ^2^
Neurologic or neuromuscular	5 (22%)	19 (18%)	0.8
Cardiovascular	8 (35%)	14 (14%)	0.029
Gastrointestinal	8 (35%)	13 (13%)	0.025
Congenital or genetic	7 (30%)	8 (7.8%)	0.007
Respiratory	6 (26%)	9 (8.7%)	0.031
Renal	1 (4.3%)	2 (1.9%)	0.5
Neonatal	1 (4.3%)	2 (1.9%)	0.5
Hematologic or immunologic	1 (4.3%)	1 (1.0%)	0.3
Malignancy	0 (0%)	1 (1.0%)	>0.9
Metabolic	0 (0%)	1 (1.0%)	>0.9
Transplant	0 (0%)	0 (0%)	>0.9

^1^ n (%); ^2^ Fisher’s exact test.

**Table 3:** Patient clinical characteristics by SPPC consult status.

Characteristic	SPPC Consult ^a,b^ N = 23	No SPPC Consult ^a,b^ N = 103	*p*-Value ^b,c^
ICU length of stay	22 (7, 56)	5 (3, 10)	<0.001
Hospital length of stay	36 (7, 59)	6 (3, 15)	<0.001
Mechanical vent days	19 (5, 36)	4 (2, 8)	<0.001
Unsuccessful extubation attempt			0.018
Yes	6 (32%)	8 (9.2%)	
No	13 (68%)	79 (91%)	
Missing	4	16	
Invasive procedures during admission			
ECMO	5 (22%)	6 (5.8%)	0.029
EVD placement	0 (0%)	2 (1.9%)	>0.9
Dialysis	0 (0%)	0 (0%)	>0.9
Chest tube	1 (4.3%)	6 (5.8%)	>0.9
ICD implantation	0 (0%)	12 (12%)	0.12
Cardiac cath	2 (8.7%)	9 (8.7%)	>0.9
LVAD placement	1 (4.3%)	1 (1.0%)	0.3
Transplant	1 (4.3%)	1 (1.0%)	0.3
Neurosurgical procedure	0 (0%)	4 (3.9%)	>0.9
New technology dependence			
Tracheostomy placed	5 (22%)	3 (2.9%)	0.005
Surgical feeding tube placed	9 (39%)	11 (11%)	0.002
Pacemaker/ICD	0 (0%)	11 (11%)	0.2
Surgical central line: PICC, Broviac, or port	1 (4.3%)	1 (1.0%)	0.3
LVAD	0 (0%)	1 (1.0%)	>0.9
Hemodialysis via HD catheter	1 (4.3%)	0 (0%)	0.2
In-hospital mortality			0.5
Death	9 (39%)	51 (50%)	
Alive	14 (61%)	52 (50%)	
Disposition			0.4
Death	9 (39%)	51 (50%)	
Home	9 (39%)	38 (37%)	
Rehab	5 (22%)	11 (11%)	
SNF	0 (0%)	3 (2.9%)	
Change in code status during admission			0.2
Yes	11 (48%)	35 (34%)	
No	12 (52%)	68 (66%)	

^a^ n (%); median (Q1, Q3). ^b^ Missing values were excluded from percentage calculations and statistical tests; ^c^ Fisher’s exact test; Wilcoxon rank-sum test.

**Table 4:** Multivariable Firth’s logistic regression model predicting SPPC consult (yes).

Variable	Odds Ratios	CI	*p*-Value
Race: Black or African American (ref. = White)	20.36	4.27–97.12	<0.001
Race: Other (ref. = White)	4.84	1.04–22.54	0.046
Number of complex chronic conditions	1.78	1.06–2.97	0.028
Previous SPPC involvement: Yes	4.23	0.51–34.71	0.197
Observations	100		

**Table 5:** SPPC characteristics among those with an SPPC consult during admission.

Characteristic	Overall ^a^ N = 23
Time from admission to SPPC consult (days)	8 (2, 13)
Time from first SPPC consult to death/discharge (days)	20 (2, 44)
Time from last SPPC consult to death/discharge (days)	1.00 (0.00, 2.00)
Goal of care documented in initial SPPC consult note	
Life prolong/focus on cure	3 (13%)
Life prolong/focus on comfort	12 (52%)
Comfort only	8 (35%)
Reason for SPPC consult (multiple choices apply)	
Diagnostic/prognostic understanding	3 (13%)
Symptom management	3 (13%)
Goals of care	22 (96%)
Medical decision making	19 (83%)
Psychosocial support	17 (74%)

^a^ Median (Q1, Q3); n (%).

## Data Availability

The original contributions presented in this study are included in the article. Further inquiries can be directed to the corresponding author.

## References

[1] Lasa J.J., Dhillon G.S., Duff J.P., Hayes J., Kamath-Rayne B.D., Levy A., Mahgoub M., Morgan R.W., McCormick T., Roberts J.S. (2025). Part 8: Pediatric Advanced Life Support: 2025 American Heart Association and American Academy of Pediatrics Guidelines for Cardiopulmonary Resuscitation and Emergency Cardiovascular Care. Circulation.

[2] Atkins D.L., Everson-Stewart S., Sears G.K., Daya M., Osmond M.H., Warden C.R., Berg R.A. (2009). Resuscitation Outcomes Consortium Investigators. Epidemiology and outcomes from out-of-hospital cardiac arrest in children: The Resuscitation Outcomes Consortium Epistry-Cardiac Arrest. Circulation.

[3] Fink E.L., Prince D.K., Kaltman J.R., Atkins D.L., Austin M., Warden C., Hutchison J., Daya M., Goldberg S., Herren H. (2016). Unchanged pediatric out-of-hospital cardiac arrest incidence and survival rates with regional variation in North America. Resuscitation.

[4] Jayaram N., McNally B., Tang F., Chan P.S. (2015). Survival After Out-of-Hospital Cardiac Arrest in Children. J. Am. Heart Assoc..

[5] Daya M.R., Schmicker R.H., Zive D.M., Rea T.D., Nichol G., Buick J.E., Brooks S., Christenson J., MacPhee R., Craig A. (2015). Out-of-hospital cardiac arrest survival improving over time: Results from the Resuscitation Outcomes Consortium (ROC). Resuscitation.

[6] Martin S.S., Aday A.W., Almarzooq Z.I., Anderson C.A.M., Arora P., Avery C.L., Baker-Smith C.M., Barone Gibbs B., Beaton A.Z., Boehme A.K. (2024). 2024 Heart Disease and Stroke Statistics: A Report of US and Global Data From the American Heart Association. Circulation.

[7] Coute R.A., Nathanson B.H., DeMasi S., Mader T.J., Kurz M.C. (2023). CARES Surveillance Group*. Disability-Adjusted Life Years Due to Pediatric Out-of-Hospital Cardiac Arrest in the United States: A CARES Surveillance Group Study. Circ. Cardiovasc. Qual. Outcomes.

[8] Meert K.L., Donaldson A.E., Newth C.J.L., Harrison R., Berger J., Zimmerman J., Anand K.J., Carcillo J., Dean J.M., Willson D.F. (2010). Complicated grief and associated risk factors among parents following a child’s death in the pediatric intensive care unit. Arch. Pediatr. Adolesc. Med..

[9] Suttle M., Hall M.W., Pollack M.M., Berg R.A., McQuillen P.S., Mourani P.M., Sapru A., Carcillo J.A., Startup E., Holubkov R. (2022). Complicated Grief, Depression and Post-Traumatic Stress Symptoms Among Bereaved Parents following their Child’s Death in the Pediatric Intensive Care Unit: A Follow-Up Study. Am. J. Hosp. Palliat. Care.

[10] Meert K.L., Slomine B.S., Christensen J.R., Telford R., Holubkov R., Dean J.M., Moler F.W. (2016). Therapeutic Hypothermia after Pediatric Cardiac Arrest Trial Investigators. Family Burden After Out-of-Hospital Cardiac Arrest in Children. Pediatr. Crit. Care Med..

[11] American Academy of Pediatrics. Committee on Bioethics and Committee on Hospital Care (2000). Palliative care for children. Pediatrics.

[12] Truog R.D., Campbell M.L., Curtis J.R., Haas C.E., Luce J.M., Rubenfeld G.D., Rushton C.H., Kaufman D.C., American Academy of Critical Care Medicine (2008). Recommendations for end-of-life care in the intensive care unit: A consensus statement by the American College [corrected] of Critical Care Medicine. Crit. Care Med..

[13] O’Keefe S., Maddux A.B., Bennett K.S., Youngwerth J., Czaja A.S. (2021). Variation in Pediatric Palliative Care Allocation Among Critically Ill Children in the United States. Pediatr. Crit. Care Med..

[14] Delgado-Corcoran C., Wawrzynski S.E., Bennett E.E., Green D., Bodily S., Moore D., Cook L.J., Olson L.M. (2020). Palliative Care in Children With Heart Disease Treated in an ICU. Pediatr. Crit. Care Med..

[15] Trowbridge A., Walter J.K., McConathey E., Morrison W., Feudtner C. (2018). Modes of Death Within a Children’s Hospital. Pediatrics.

[16] Gans D., Kominski G.F., Roby D.H., Diamant A.L., Chen X., Lin W., Hohe N. (2012). Better outcomes, lower costs: Palliative care program reduces stress, costs of care for children with life-threatening conditions. Policy Brief. UCLA Cent. Health Policy Res..

[17] Linebarger J.S., Johnson V., Boss R.D. (2022). The Section on Hospice and Palliative Medicine. Guidance for Pediatric End-of-Life Care. Pediatrics.

[18] Gouda S.R., Snaman J.M., D’Anna R., Upham E.J., Dahlberg S.E., Rosenberg A.R., DeCourcey D.D. (2025). Pediatric Palliative Care Consultation in the PICU Following Out-of-Hospital Cardiac Arrest: Analysis of the U.S. Pediatric Health Information Systems Database. Pediatr. Crit. Care Med..

[19] Gouda S.R., Bohr N.L., Hoehn K.S. (2022). Palliative Care Utilization Following Out-of-Hospital Cardiac Arrest in Pediatrics. Crit. Care Explor..

[20] Feinstein J.A., Hall M., Davidson A., Feudtner C. (2024). Pediatric Complex Chronic Condition System Version 3. JAMA Netw. Open.

[21] Johnston E.E., Bogetz J., Saynina O., Chamberlain L.J., Bhatia S., Sanders L. (2019). Disparities in Inpatient Intensity of End-of-Life Care for Complex Chronic Conditions. Pediatrics.

[22] Currie E.R., Wolfe J., Boss R., Johnston E.E., Paine C., Perna S.J., Buckingham S., McKillip K.M., Li P., Dionne-Odom J.N. (2023). Patterns of Pediatric Palliative and End-of-Life Care in Neonatal Intensive Care Patients in the Southern U.S. J. Pain Symptom Manag..

[23] Muni S., Engelberg R.A., Treece P.D., Dotolo D., Curtis J.R. (2011). The influence of race/ethnicity and socioeconomic status on end-of-life care in the ICU. Chest.

[24] Lutmer J.E., Humphrey L., Kempton T.M., Moore-Clingenpeel M., Ayad O. (2016). Screening Criteria Improve Access to Palliative Care in the PICU. Pediatr. Crit. Care Med..

[25] Holder P., Coombes L., Chudleigh J., Harding R., Fraser L. (2024). Barriers and facilitators influencing referral and access to palliative care for children and young people with life-limiting and life-threatening conditions. Palliat. Med..

